# ATTfold: RNA Secondary Structure Prediction With Pseudoknots Based on Attention Mechanism

**DOI:** 10.3389/fgene.2020.612086

**Published:** 2020-12-15

**Authors:** Yili Wang, Yuanning Liu, Shuo Wang, Zhen Liu, Yubing Gao, Hao Zhang, Liyan Dong

**Affiliations:** ^1^College of Software, Jilin University, Changchun, China; ^2^Key Laboratory of Symbolic Computation and Knowledge Engineering of Ministry of Education, Jilin University, Changchun, China; ^3^College of Computer Science and Technology, Jilin University, Changchun, China; ^4^Graduate School of Engineering, Nagasaki Institute of Applied Science, Nagasaki, Japan

**Keywords:** RNA, secondary structure prediction, pseudoknot, attention mechanism, deep learning, hard constraints

## Abstract

Accurate RNA secondary structure information is the cornerstone of gene function research and RNA tertiary structure prediction. However, most traditional RNA secondary structure prediction algorithms are based on the dynamic programming (DP) algorithm, according to the minimum free energy theory, with both hard and soft constraints. The accuracy is particularly dependent on the accuracy of soft constraints (from experimental data like chemical and enzyme detection). With the elongation of the RNA sequence, the time complexity of DP-based algorithms will increase geometrically, as a result, they are not good at coping with relatively long sequences. Furthermore, due to the complexity of the pseudoknots structure, the secondary structure prediction method, based on traditional algorithms, has great defects which cannot predict the secondary structure with pseudoknots well. Therefore, few algorithms have been available for pseudoknots prediction in the past. The ATTfold algorithm proposed in this article is a deep learning algorithm based on an attention mechanism. It analyzes the global information of the RNA sequence via the characteristics of the attention mechanism, focuses on the correlation between paired bases, and solves the problem of long sequence prediction. Moreover, this algorithm also extracts the effective multi-dimensional features from a great number of RNA sequences and structure information, by combining the exclusive hard constraints of RNA secondary structure. Hence, it accurately determines the pairing position of each base, and obtains the real and effective RNA secondary structure, including pseudoknots. Finally, after training the ATTfold algorithm model through tens of thousands of RNA sequences and their real secondary structures, this algorithm was compared with four classic RNA secondary structure prediction algorithms. The results show that our algorithm significantly outperforms others and more accurately showed the secondary structure of RNA. As the data in RNA sequence databases increase, our deep learning-based algorithm will have superior performance. In the future, this kind of algorithm will be more indispensable.

## 1. Introduction

RNA is an indispensable biopolymer that plays diverse biological roles in regulating translation (Kapranov et al., 2007), gene expression (Storz and Gottesman, 2006), and RNA splicing (Sharp, 2009). The sophisticated 3-dimensional shape (tertiary structure) of RNA is the cornerstone of its functions (Ding et al., 2014), which allows RNA to exert various activities (Tinoco and Bustamante, 1999). It is relatively easy and feasible to predict the RNA secondary structure using RNA sequence data, but it is quite difficult to accurately predict the RNA tertiary structure. To accurately obtain the RNA secondary structure, different prediction algorithms have been developed over the past 40 years.

The prediction of the RNA secondary structure is a subject that has been extensively studied for a long time, but there are still many problems. First, the most accurate and reliable approach is to directly observe and obtain the RNA secondary structure via chemical and physical methods, including X-ray crystal diffraction and NMR biological experiments, but this approach is particularly time-consuming, laborious, and expensive (Novikova et al., 2012) and is thus not suitable for large-scale predictions. Notably, the most mainstream calculation method is the Nearest Neighbor Thermodynamic Model (NNTM) based on a single RNA sequence (Turner and Mathews, 2010). This method calculates the RNA secondary structure with minimum free energy (MFE) through the dynamic programming algorithm. Moreover, the more prominent algorithm tools based on the above algorithms are RNAfold (Zuker and Stiegler, 1981), mfold (Zuker, 2003), and RNAstructure (Reuter and Mathews, 2010). The idea of the MFE algorithm is to enumerate all possible structures and to then elucidate the structure with MFE. However, the number of enumerated structures increases exponentially with the increase in RNA sequence length, making it difficult to predict the structure of a long sequence. In addition, two distinctly different RNA secondary structures may show similar energy, which greatly reduces their accuracy. As for classic algorithms, they only focus on the number of pairing bases in the sequence, while ignoring the exact base pairs. Although such algorithms perform well in terms of the prediction accuracy, they deliver poor reports to describe the true RNA secondary structures. As a result, it is impossible to intuitively learn about the functions or biological roles of RNA. In the meantime, none of these algorithms predict the RNA secondary structure with pseudoknots. At present, the thermodynamic matcher is still a very general framework used to solve the hard constraints of RNA secondary structure (Reeder and Giegerich, 2004).

With the rapid growth of deep learning, a large number of topics have been revisited and great breakthroughs have been made, including the prediction of the RNA secondary structure. The main cause for its rapid development and success is that deep learning methods further optimize network parameters by training mass data, so as to extract the hidden features from data not observed and calculated by human resources. After further identification of the hidden features, a corresponding prediction model is constructed. Finally, using this model, the real and effective structure can be predicted. The CD fold algorithm proposed in Zhang et al. (2019) calculates the MFE of the RNA secondary structure by the convolutional neural network combined with the dynamic programming (DP) algorithm. Additionally, the algorithm put forward in Willmott et al. (2020) combines the recurrent neural network with the SHAPE soft constraints. The e2efold algorithm proposed in Chen et al. (2020) is trained on the large-scale training sets with the use of an end-to-end model. Notably, the above-mentioned algorithms greatly outperform the traditional ones. Furthermore, it has been suggested in numerous studies that, the pseudoknot structure in the RNA secondary structure greatly affects the biological functions and is found in diverse kinds of RNA (Brierley et al., 2007), like transfer messenger RNA (tmRNA), ribosomal RNA (rRNA), and viral RNA. On the other hand, the pseudoknot exhibits great activities in translation regulation, ribosomal frame shifting, and RNA splicing. Therefore, it is an indispensable step to predict the RNA pseudoknot structure, which better reveals the real and effective RNA secondary structure and sheds more light on the various functions of RNA.

In this article, ATTfold, a deep learning algorithm, was proposed to precisely predict the RNA secondary structure with pseudoknots. In ATTfold, tens of thousands of RNA sequences from multiple families along with their real secondary structure data were trained to enhance the algorithm robustness. By combining the unique form of hard constraints of RNA secondary structure, the proposed algorithm predicted the RNA secondary structures with or without pseudoknots in different RNA families. Additionally, it more effectively solved the prediction problem for long RNA sequences based on the characteristics of the attention mechanism.

## 2. Data and Methods

### 2.1. Database Selection

As is known, mass data are generally required for work based on deep learning algorithms, but the number of RNA sequences in some previous databases commonly used to predict RNA secondary structure cannot meet these needs [for example, RNA STRAND (Andronescu et al., 2008) has 4,666 RNA sequences, while ArchiveII (Sloma and Mathews, 2016) has 3,975 RNA sequences]. In this article, the RNAStralign Database (Tan et al., 2017), which altogether contains 37,149 RNA sequences, was used.

### 2.2. Calculation Method

As suggested by the name, the RNA secondary structure is a two-dimensional figure, mainly constituted by complementarily paired bases and unpaired bases in one-dimensional RNA sequence. Therefore, the real RNA secondary structure can be obtained, so long as the positions of all paired bases in the RNA sequence are determined. The traditional dot bracket representation is a one-dimensional structure that can well represent RNA secondary structure without pseudoknots, but not with pseudoknots. In this study, an N*N symmetric matrix was used (N is the sequence length), where the horizontal coordinates i and vertical coordinates j, indicated the position of the base in the RNA sequence. Using this matrix, the actual base pairing condition of various RNA secondary structures can be fully displayed. Furthermore, this matrix can also be used to predict the RNA secondary structures with or without pseudoknots for the various problems of the traditional RNA secondary structure prediction methods mentioned in this article. Subsequently, a more sophisticated model called ATTfold was proposed to solve the problems.

**Figure 1:**
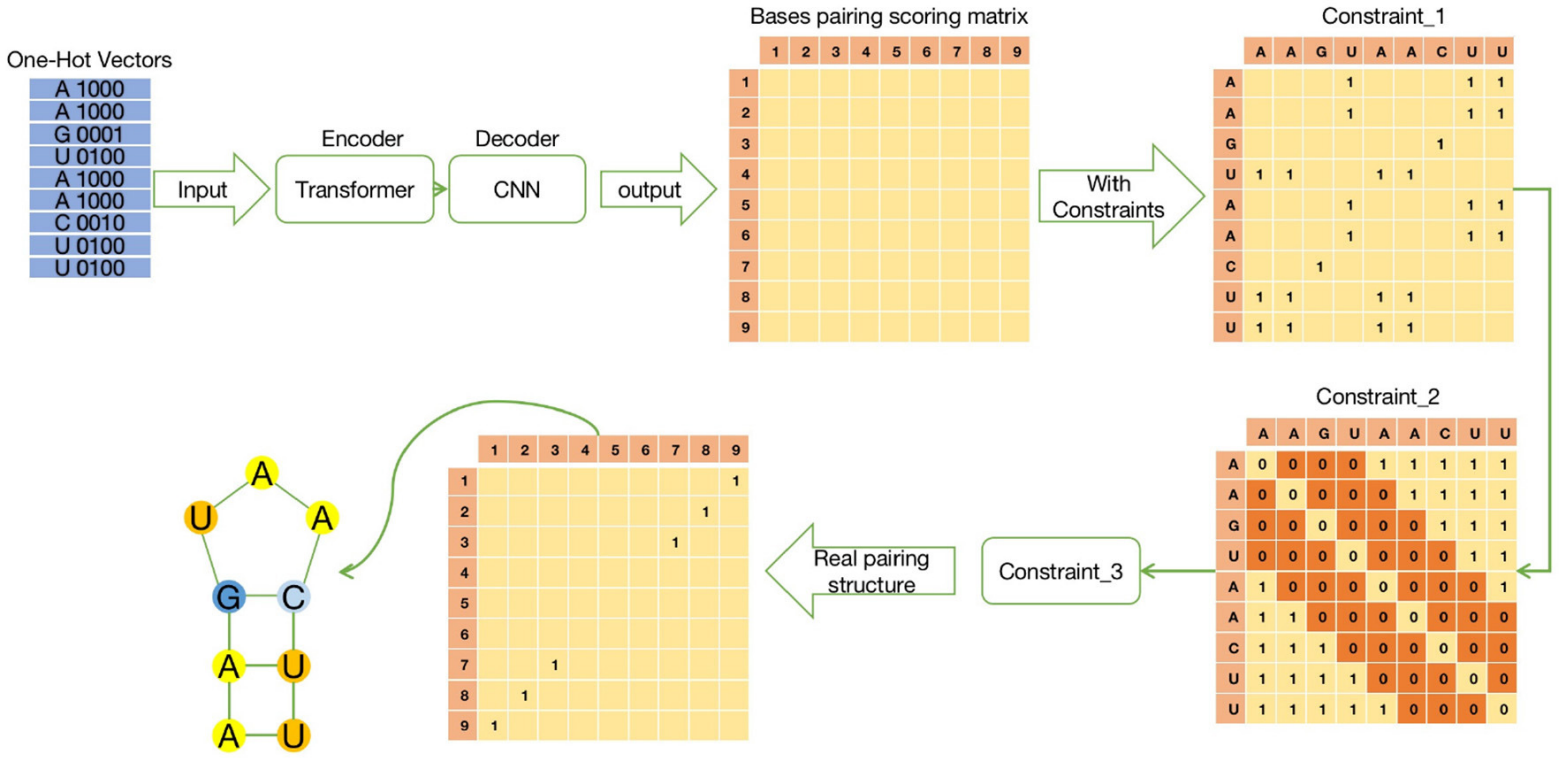
The schematic diagram of ATTfold architecture, which contains three parts: Transformer, CNN, and Constrain. Transformer is an encoder network, mainly responsible for recoding the input One-Hot vectors of RNA sequences. CNN is a decoder network to predict the bases pairing scoring matrix. Constrain has three constraints to constrain the bases pairing scoring matrix and get the real secondary structure.

ATTfold was a deep learning model constructed by combining the attention mechanism with the hard constraints of the RNA secondary structure. Its overall structure was divided into three parts, namely, Encoder-Decoder-Constrain. The specific process is shown in Figure 1. The encoder used the transformer model with an attention mechanism (Vaswani et al., 2017), which performed recording on the one-hot encoding of the input RNA sequence and trained this encoding information, so as to obtain more hidden features of the RNA sequence. The decoder used the convolutional neural network model. By convolving the output obtained by the encoder training, an N*N symmetric bases pairing score matrix was eventually acquired. In this bases pairing scoring matrix, the score of each position (i, j) represented the probability of base i pairing with base j. The bases pairing scoring matrix obtained through the “encoder-decoder” part was an unconstrained matrix, which might lead to a probability of pairing in every position, including base pairing with itself.

Therefore, the RNA secondary structure formed by this bases pairing scoring matrix was not a real and effective structure. To obtain a true and effective RNA secondary structure from the output of this network, the constraints that the RNA sequence must comply with when forming its secondary structure should be added, which are also called hard constraints. Lastly, after the bases pairing scoring matrix was hard-constrained, the corresponding bases pairing matrix was obtained. Each position in the matrix contained only two numbers 0 or 1, where 0 meant that i, j bases were not paired, whereas 1 meant that they were paired.

#### 2.2.1. RNA Sequence to the Bases Pairing Scoring Matrix

The ATTfold model was unable to directly recognize and calculate characters (A, U, G, C) of the RNA sequence or the pairing structure. Therefore, they should be encoded and represented, respectively. The RNA sequence character encoding is presented in Table 1. The (N*4) one-hot encoding was performed for the entire RNA sequence in this study. However, as we know, one-hot encoding is not perfect in expressing the hidden features of each base, and a 4-dimensional feature cannot fully express the hidden features of RNA sequences. Therefore, the one-hot encoding was converted into d-dimensional (N*d, d>4) coding through conv1d, which was then used in combination with the transformer model according to the attention mechanism; in this way, the RNA sequence was recorded. The above operations were used to acquire more useful hidden information. Additionally, due to the attention mechanism, ATTfold was able to better handle the long RNA sequences. The principle is as follows:

(1) $$\begin{array}{r} Attention\left(Q,K,V\right)=softmax\left(\frac{QK^{T}}{\sqrt{d}}\right)V \end{array}$$

Among them, Query (Q), KEY (K), and Value (V) were all obtained through the coding matrix (N*d) via the respective linear transformation matrix, and the Attention value represented the degree of correlation between bases. In other words, it represented the size of the role of all bases in the RNA sequence when one base was paired or unpaired with another one. Consequently, the model obtained more hidden features related to the RNA bases. In addition, the input method of the transformer model was not in the same order as the Recurrent Neural Network model, so that it was impossible to distinguish the position of each base in the RNA sequence, thus losing some important information. To solve this problem, the concept of position embedding was innovatively introduced into the transformer model and the corresponding position embedding information was also introduced into our ATTfold, as shown below:

(2) $$\begin{array}{r} POS\text{\_}bas=1,2,\ldots ,N \end{array}$$

(3) $$\begin{array}{r} POS\text{\_}rel=1/N,2/N,\ldots ,1 \end{array}$$

Among them, Pos_bas(N*1) and Pos_rel(N*1) were the absolute and relative positions of the base, respectively. To further obtain the relevant position information and to facilitate the merging of position information with input information, the absolute position was combined with the relative position by a linear transformation method and converted into the position embedding matrix (N*d), which was consistent with the dimension of the sequence information. In this way, a complete input coding was obtained through the matrix addition of position embedding information with sequence encoding information.

**Table 1:** The rules of transformation between bases and One-Hot vectors.

**Input bases**	**One-Hot encoding**
A	[1,0,0,0]
U	[0,1,0,0]
C	[0,0,1,0]
G	[0,0,0,1]
N	[0,0,0,0]

To obtain the structure information of the pseudoknot and to facilitate our calculation of the loss function and model accuracy in deep learning, the corresponding symmetric matrix (N*N) of the known RNA sequence structure was encoded, since encoding the dot bracket representation of the traditional method no longer met our needs. The horizontal coordinates and vertical coordinates indicated the absolute position of each base in the RNA sequence. Each row and column, which represented the specific position of the i base and j base pairing, was at most 1, while the rest were 0. In this way, RNA secondary structures with and without pseudoknots were well displayed at the same time.

**Figure 2:**
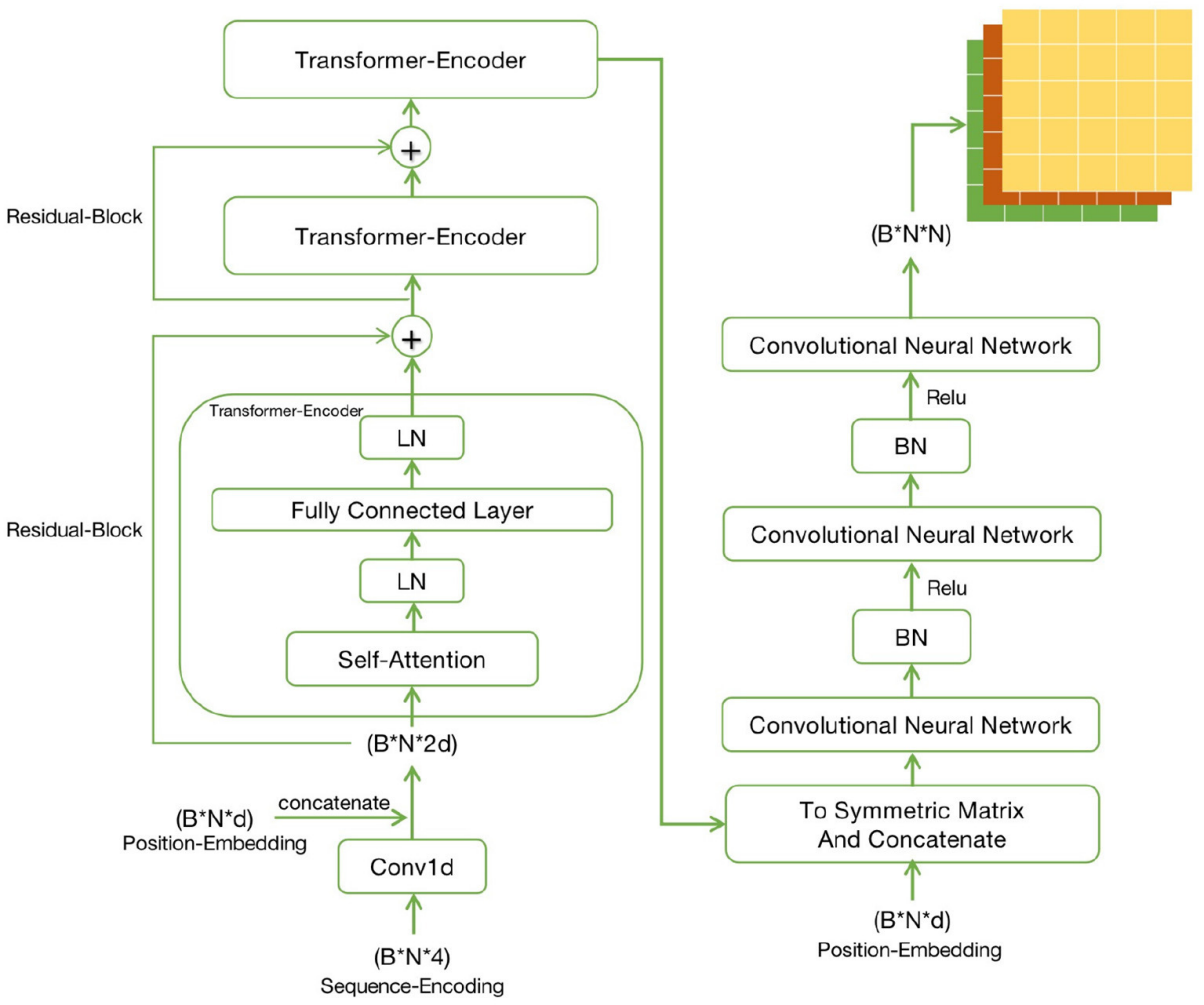
Detailed diagram of Encoder network and Decoder network.

The entire Encoder-Decoder network structure is displayed in Figure 2. As for the Encoder network, its input consisted of two parts, namely, the Sequence-Encoding (B*N*4) and Position-Embedding (B*N*d), where B (Batch size) represents the number of RNA sequences input to the network each time, N is the sequence length and d is the characteristic dimension. The input encoding was first trained by three identical transformer models using the attention mechanism. Then, to solve the problems of gradient disappearance and local extremum during the training of deep learning network, Residual-Block was also introduced. The training encoding should be normalized in the transmission process, so as to limit the value within a certain range, thereby eliminating the adverse effects induced by singular sample data. The Layer-Normalization (LN) (Ba et al., 2016) was more effective at dealing with the sequence problems.

Regarding the Decoder, its input was a symmetric matrix formed by processing the output of the Encoder and Position-Embedding. The symmetric matrix passed through three different Convolutional Neural Networks, finally forming the symmetric bases pairing scoring matrices (B*N*N). Similarly, the data should still be normalized in the process of processing. The matrix (similar image) data were processed, so Batch-Normalization (BN) (Ioffe and Szegedy, 2015) was more effective. But the true and effective RNA secondary structure might not be obtained through the bases pairing scoring matrix, so it should be constrained. Finally, this matrix that combined the hard constraints of RNA secondary structure was able to predict a real secondary structure.

#### 2.2.2. Hard Constraints Representation

Hard constraints represent a principle that each RNA secondary structure must comply with, and are mainly used for RNA folding. Soft constraints are the additional pseudo-energy terms used by most new methods, which stands for a reward and punishment mechanism implemented during the bases pairing process (Lorenz et al., 2016). As for ATTfold, only the hard constraints were adopted to predict the RNA secondary structure, since the deep learning model itself contained a reward and punishment mechanism. Using the back-propagation algorithm, the corresponding parameters were updated. The following three items were included in the hard constraints:

1. RNA bases pairing only satisfied A-U and G-C Watson-Crick pairs (Watson and Crick, 1953), and G-U Wobble pair (Varani and McClain, 2000);
2. In the case of RNA bases pairing, the distance between two bases should be greater than 3, because the formed hairpin loops must contain at least 3 bases;
3. In the case of RNA bases pairing, the base was only paired once with other bases.

For the above three hard constraints, corresponding solutions were also proposed. First, this study aimed to constrain the bases pairing scoring matrix of the last output in the previous network, which conformed to the real RNA bases pairing structure. Therefore, the constraints should be converted into a symmetric matrix to facilitate calculations.

For constraint 1, bases pairing constraints should be performed for each input sequence. Then, the input sequence was converted into an N*N symmetric matrix, where the rows and columns were the absolute positions of the bases, the positions matched with the A-U, G-C, and G-U pairs were filled with 1, while the rest were 0. Afterwards, matrix multiplication of Constraint_1 matrix and the bases pairing scoring matrix was carried out. For positions that met the conditions, the predicted values were retained, while positions that did not meet the condition were returned to 0, as shown in Constraint_1 of Figure 1.

For constraint 2, a certain base was not paired with others when the surrounding distance was <4. Therefore, it was also set as an N*N symmetric matrix like constraint 1, and its diagonal value was set to 0 (the base was not paired with itself). Later, all the values at the top, bottom, left, and right were set to 0, for which the distance to the diagonal was set to 3, while the remaining values were set to 1. In this way, the condition of constraint 2 was satisfied, and the specific form was defined as Constraint_2 in Figure 1.

For constraint 3, even though the above two constraints were performed on the bases pairing scoring matrix and the sparse of matrix was made on a large scale, there was still a one-to-many bases pairing situation. In this case, the DP algorithm was not suitable, even though it was able to obtain the optimal solution, since this article focused on the prediction problem of long sequences. To facilitate the calculation, constraint 3 was made through the following simplified operations:

(4) $$\begin{array}{r} relu\left(Sn-1\right)=0,n=1,\ldots ,N \end{array}$$

*Sn* represented the number of pairs in each base, and the meaning in the matrix indicated the sum of the values of each row or column. From constraint 3, *Sn* ≤ 1 was observed. Therefore, it was transformed into the above formula, which well-expressed the effect of constraint 3. So far, the three hard constraints have been successfully transformed into a single constraint problem. For the optimization algorithm for a single constraint problem, the Lagrange Multiplier Method was utilized to solve this problem perfectly (Chen et al., 2020). Compared with the DP algorithm, this algorithm substantially reduced the calculation time and improved the prediction accuracy.

Through the synthesis of the above conditions, the final objective function of ATTfold was obtained, as shown below:

(5) $$\begin{array}{r} \max\frac{1}{2}\underset{part\;1}{\underset{︸}{\left(S-s\right)*R}}+\underset{part\;2}{\underset{︸}{w*relu\left(S_{n}-1\right)}}+\underset{part\;3}{\underset{︸}{\rho ∥R∥}} \end{array}$$

(6) $$\begin{array}{r} \min-\left(\frac{1}{2}\left(S-s\right)*R+w*relu\left(S_{n}-1\right)+\rho ∥R∥\right) \end{array}$$

**Figure 3:**
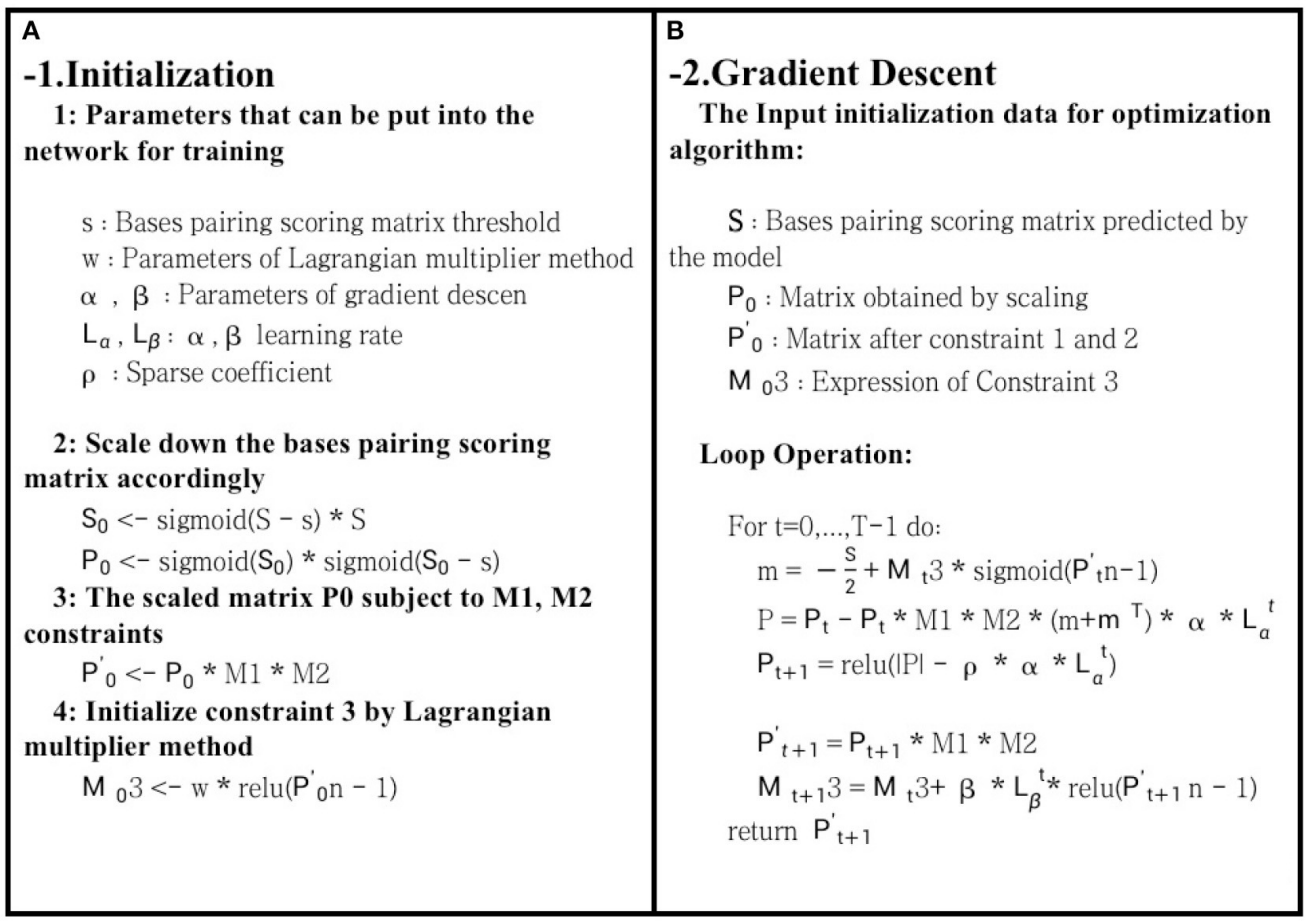
Schematic diagram of bases pairing scoring matrix optimization. The role of **(A)** is to initialize each parameter and variable during the optimization process. **(B)** uses the gradient descent optimization algorithm to optimize the bases pairing scoring matrix. Finally, it returns a two-dimensional matrix that meets the hard constraints of RNA secondary structure.

*S* represented the bases pairing score matrix predicted, *R* was the real structure matrix, *Sn* referred to the sum of the values in each row of *S*, and ρ||*R*|| indicated the L1 regularization penalty term, which was used to improve matrix sparsity and prevent overfitting. However, *s* represented a threshold. In the case of *Sij* > *sij*, bases i and j were paired, and vice versa. Part 1 of formula (5) was the main part of the entire function, and the degree of fit between the predicted structure and the real structure was obtained. Furthermore, part 2 was constraint 3, while part 3 was the L1 regularization penalty. The goal of this article was to find the maximum fit between the predicted and the real structures, and it was inconvenient for us to optimize it. Therefore, to optimize the maximum fit using the gradient descent method, formula (6) was used as our final objective function. The optimization process is shown in Figure 3. After optimization for T times by the gradient descent method, the final matrix obtained was our final prediction matrix.

The secondary structure formed by the prediction matrix obtained for the first time is far from the real secondary structure. With a large amount of labeled data, we also need to optimize each parameter in the ATTfold model through the backpropagation algorithm. Finally, the network trained through the training set, with the help of the validation set, finds a set of parameters for the optimal result as the parameters of the final model. Lastly, the final prediction matrix obtained by ATTfold can be closer to the real secondary structure. During this process, in order to correspond to the final prediction matrix and to better optimize the algorithm, we also converted the real RNA secondary structure into the corresponding matrix form as the final label.

## 3. Results

**Figure 4:**
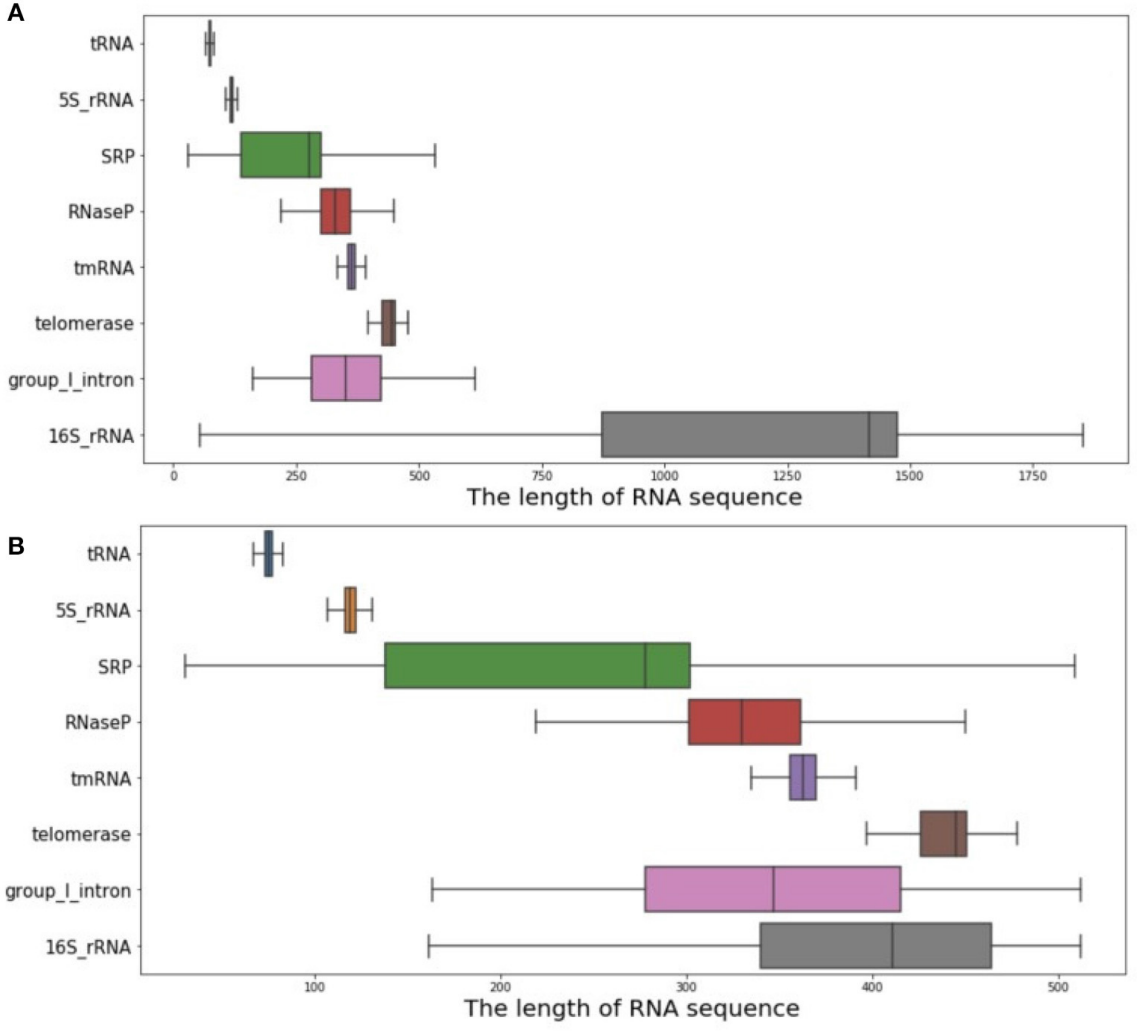
RNA sequence length analysis chart. **(A)** shows the distribution of the length of all RNA sequences of the eight RNA families. **(B)** shows the distribution of RNA sequence length within 512.

As introduced above, a database including 37,149 RNA sequences and their real structures was used as the experimental database in this article. It contains eight RNA families, including 5S_rRNA, tRNA, group_1_intron, 16S_rRNA, tmRNA, SRP, RNaseP, and telomerase. Through data set analysis, the length distribution of the RNA sequence was particularly uneven, due to the wide variety of RNA families contained in the data set. Typically, 16S_rRNA was the family with the greatest impact, but its RNA sequence length was too long, along with a relatively large amount of data, eventually leading to the above problems. As shown in Figure 4A, if all such data were used as the input data of ATTfold, it might greatly increase the difficulty in model training, making it difficult to find most of the effective hidden features. Therefore, to prevent the condition that the particularly long data affected the model but not the training and prediction of other RNA families, the RNA data set with a RNA sequence length not greater than 512, was utilized as the final experimental data. The sequence length distribution is displayed in Figure 4B. Additionally, to enhance the generalization ability of the ATTfold model, the RNA sequence data of the above-mentioned eight RNA families, with the length not exceeding 512, were trained simultaneously. A total of 25,425 RNA sequences met this condition. To better verify the model accuracy and robustness, all the RNA sequences were divided into training set, verification set, and test set, respectively. In the meantime, to avoid the condition that the same sequence data affected the final experiment, the test set was compared with the training set and the de-redundancy operation was performed. In the other words, the same sequence in the test set (which served as the training set) was deleted. The specific distribution is exhibited in Table 2. In this experiment, our network model was trained through the training set, and the model was selected through the validation set. Finally, by inputting the test set sequence into the network, the prediction result was obtained and compared with the real structure, so as to measure the generalization ability of the entire model.

**Table 2:** RNAStralign dataset statistics.

**RNA type**	**Length**	**All_Num**	**512_Num**	**Train**	**Val**	**Test**	**Deredundancy**
5S_rRNA	104–132	11419	11419	9172	1114	1133	867
tRNA	59-95	9245	9245	7405	933	907	527
group_1_intron	163–615	2135	2058	1606	237	215	116
16S_rRNA	54–1851	12608	973	765	101	107	84
tmRNA	102–437	637	637	519	60	58	49
SRP	30–553	601	591	480	45	66	48
RNaseP	189–486	467	467	363	52	52	45
telomerase	382–559	37	35	30	1	4	4
Total	30–1851	37149	25425	20340	2543	2542	1740

In this chapter, the prediction results of the ATTfold method were elaborated and compared with four traditional classic algorithms [namely, RNAfold (Zuker and Stiegler, 1981), mfold (Zuker, 2003), RNAstructure (Bellaousov et al., 2013), Probknot (Bellaousov and Mathews, 2010)]. These four methods have extensively been used with a high authority in this field, all of which have their own websites for the online prediction of the RNA secondary structure, which is very convenient for users. Among these four methods, the methods of predicting the secondary structure with and without pseudoknots were included, which were also compared with the ATTfold algorithm to better verify our model performance. To unify the evaluation standard and to make it possible to predict the real RNA secondary structure well by means of the evaluation standard, a two-dimensional symmetric matrix (N*N) was employed to simultaneously represent the known and the predicted RNA secondary structures (where n represented the length of each sequence). In this two-dimensional symmetric matrix, 0 at each position indicated that two bases were not paired, while 1 indicated that two bases were paired. Unlike the traditional (1*N) one-dimensional matrix, 0 in the matrix suggested that the base was not paired, whereas 1 indicated that the base was paired. The traditional representation method only judges whether a base is paired, rather than the exact base that it is paired with. Therefore, the predicted secondary structure is far from the real structure, regardless of the higher score obtained.

### 3.1. Performance Metrics

To better judge the RNA secondary structure predicted by ATTfold, two evaluation criteria (a pair of contradictory performance metrics) were used, namely, sensitivity (SEN) and positive predictive value (PPV). SEN referred to the number of true paired bases predicted by ATTfold, while PPV indicated the number of true paired bases predicted by ATTfold. To have a more comprehensive evaluation, the new performance matrix composed of SEN and PPV was quoted, and named F1-score (Yonemoto et al., 2015), as follows:

(7) $$\begin{array}{r} SEN=TP/\left(TP+FN\right) \end{array}$$

(8) $$\begin{array}{r} PPV=TP/\left(TP+FP\right) \end{array}$$

(9) $$\begin{array}{r} F1-score=2*\left(\left(SEN*PPV\right)/\left(SEN+PPV\right)\right) \end{array}$$

Among them, TP, FN, FP, and TN were better represented by Table 3. TP was the number of base pairing positions in the bases pairing scoring matrix predicted by ATTfold, that were the same as the actual secondary structure base pairing positions. FN indicated the number of base pairing positions predicted by ATTfold to be 0 in the actual secondary structure. FP was the number of unpaired positions of the true secondary structure base, predicted by ATTfold to be 1. TN indicated the number of predicted unpaired positions that were the same as the true unpaired positions. There were more unpaired bases in the sequence, so TN was not used as a condition to judge the model quality. Additionally, the F1-score integrated the advantages of SEN and PPV, and better judged the model quality. Therefore, the value of the F1-score was finally used as the evaluation standard of the model.

**Table 3:** The specific representation of each parameter in the performance metrics.

		**Predict**	
		P	N
True	P	TP	FN
	N	FP	TN

### 3.2. Performance Comparison

**Figure 5:**
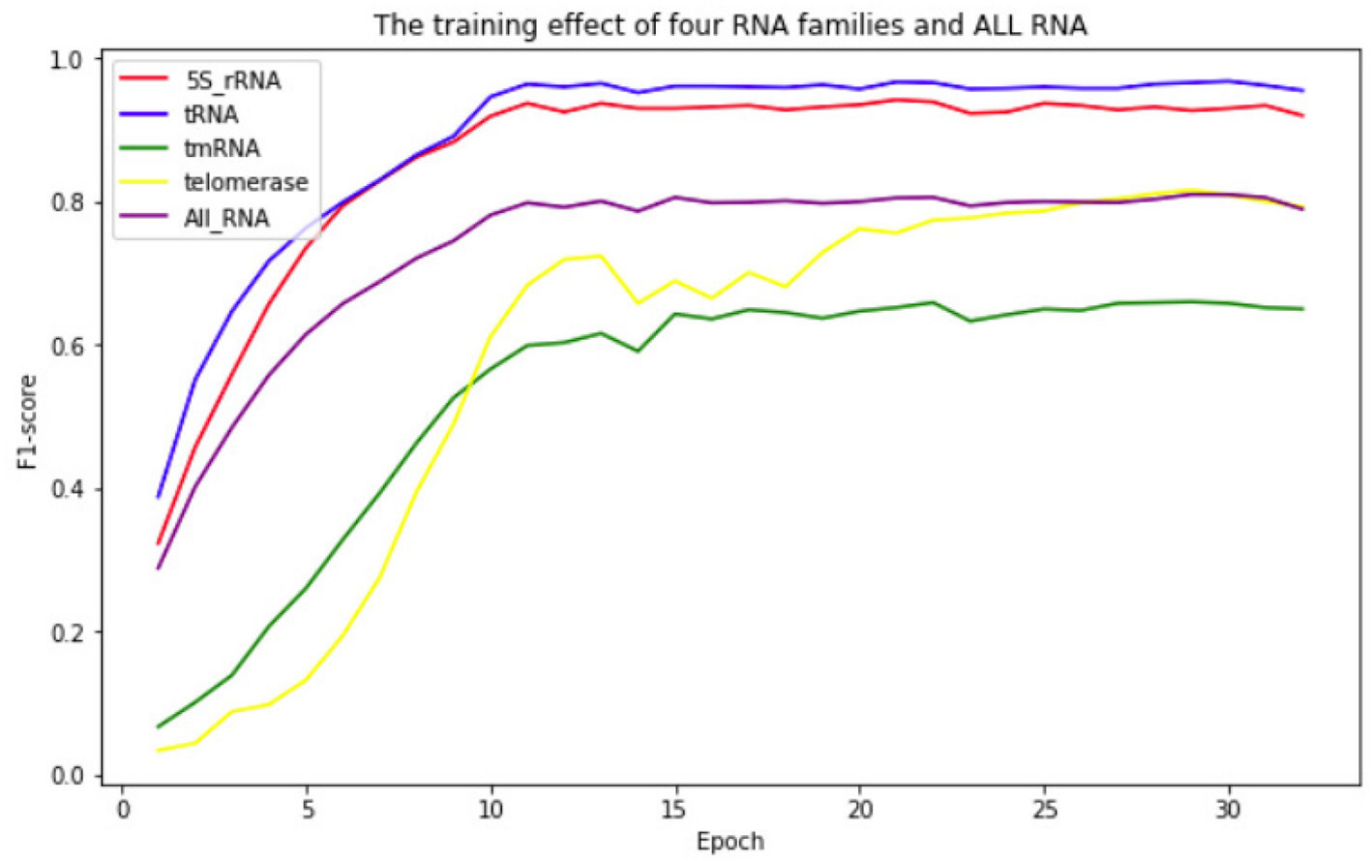
The effect of ATTfold on the prediction of 4 RNA families and all RNA sequences.

To show the fitting effect of ATTfold on the RNA secondary structures of different families more clearly, each family in the test set was evaluated separately. In the end, four families had better scoring effects–5S_rRNA, tRNA, Telomerase, and tmRNA. The fitting effect of ATTfold on these four families is presented in Figure 5. As observed, the 5S_rRNA and tRNA families had a relatively fast fitting speed, and the prediction effect entered a flat stage after 12 Epochs. Their final prediction values were stable at above 0.9, indicating that ATTfold had an excellent predictive effect on these two RNA families. For the tRNA and telomerase families, the early prediction effect rapidly increased with the increase in the training Epoch. Then, it entered a period of fluctuation, after which, the telomerase family still had a period of slow rise and was gradually stabilized at around 0.8, whereas the tmRNA family was quickly stabilized at around 0.65. In addition to the description of those prediction effects on four RNA families, the overall effect of the total RNA sequence on predicting its secondary structure was also described (purple curve). The overall prediction effect was stabilized after 15 Epochs and remained at around 0.8. The best prediction result of ATTfold for all RNA sequences was chosen as the final prediction effect (the F1-score at the 28th Epoch was 0.8097), and the corresponding model parameters were saved as the final Model. Under the optimal results, the final prediction results of these four well-performing RNA families were introduced as follows.

Table 4 shows the comparison results between ATTfold and the other four traditional algorithms in the same test set in the 5S_rRNA and tRNA families. As a result, our method displayed obvious advantages over the other four methods in evaluating these two RNA families. Compared with the highest scores of the other four traditional algorithms, the F1-socre of this method increased by 25 and 21%, respectively, and even reached a super good effect of over 90%. Such a phenomenon can be explained by two factors. First, in the training set of the entire network, the number of 5S_rRNA and tRNA RNA sequences accounted for 81.5% of the total number of sequences. In this way, the hidden features extracted by the entire network and the parameters in the network were mainly formed for these two families. Second, the RNA sequence lengths of 5S_rRNA and tRNA ranged from 59 to 132. As shown in Figure 4B, there were small differences between the longest and the shortest sequence lengths of these two families, and the overall sequence length was concentrated in a small range. In addition, the number of base pairings was relatively small. In summary, the deep learning network easily learned the corresponding pairing information of these two RNA families, so the total F1-score achieved a very high effect. However, after comparison, the scoring effect of the mfold and RNA structure methods through the two-dimensional matrix was relatively low, which did not reach the scoring height of the classic algorithm. As a result, a one-dimensional matrix score of these two RNA families was obtained for these four traditional algorithms, as shown in Table 5.

**Table 4:** Compare ATTfold and four traditional algorithms on tRNA and 5S_rRNA by using a two-dimensional matrix.

**Method**	**tRNA**			**5S_rRNA**		
	**F1-score**	**PPV**	**SEN**	**F1-score**	**PPV**	**SEN**
ATTfold	**0.966**	**0.972**	**0.961**	**0.927**	**0.933**	**0.923**
RNAfold	0.753	0.722	0.787	0.683	0.652	0.719
mfold	0.603	0.628	0.58	0.406	0.425	0.394
RNAstructure	0.619	0.604	0.637	0.458	0.44	0.478
Probknot	0.738	0.696	0.792	0.666	0.655	0.681

*The bold value is the maximum of each column*.

**Table 5:** Performance of four traditional algorithms in one-dimensional matrix.

**Method**	**tRNA**			**5S_RNA**		
	**F1-score**	**PPV**	**SEN**	**F1-score**	**PPV**	**SEN**
RNAfold	0.867	0.831	0.911	0.831	0.79	0.878
mfold	0.765	0.82	0.735	0.674	0.726	0.639
RNAstructure	0.795	0.775	0.82	0.758	0.73	0.791
Probknot	0.853	0.803	0.917	0.814	0.798	0.835

As observed in 5S_rRNA, the scores of the four traditional algorithms increased by 22% on average, while the score of each algorithm increased by 15% on average in tRNA, and these four traditional methods reached a relatively high score. Therefore, the traditional method is mainly used to predict whether the relative position base is matched but does not accurately predict the exact base that it is matched with. Accordingly, the secondary structure formed by the paired base predicted in this way is quite different from the actual secondary structure. In contrast, our ATTfold model better predicted the precise position of base-to-base pairing. Similarly, the RNA secondary structure closer to the real state was also obtained.

Apart from the above two short RNA sequence families, Table 6 shows the scoring effects of Telomerase and tmRNA–the two long RNA sequence families. In these two families with longer RNA sequences, our algorithm made great progress compared with the other four traditional algorithms, even though the overall effect predicted by our method was not as good as the first two families. The F1-score values increased by 26 and 22%, respectively, from the highest score. Compared with the other four RNA families not on the list, these two RNA families had a smaller overall RNA sequence length, as displayed in Figure 4B, making it easier for the model to find the useful hidden features. Notably, the small amount of data was responsible for the inferior prediction effect of our model to the first two RNA families. Therefore, in the future, more RNA molecules of this family should be identified, which will allow our model to have a better predictive effect on them with the continuously enlarging data volume. In this regard, there is still a lot of room for model improvement.

**Table 6:** Compare ATTfold and four traditional algorithms on Telomerase and tmRNA by using a two-dimensional matrix.

**Method**	**Telomerase**			**tmRNA**		
	**F1-score**	**PPV**	**SEN**	**F1-score**	**PPV**	**SEN**
ATTfold	**0.816**	**0.846**	**0.791**	**0.66**	**0.686**	**0.64**
RNAfold	0.556	0.485	0.652	0.406	0.385	0.433
mfold	0.442	0.392	0.507	0.442	0.392	0.507
RNAstructure	0.432	0.377	0.505	0.365	0.344	0.389
Probknot	0.474	0.418	0.548	0.435	0.41	0.465

*The bold value is the maximum of each column*.

## 4. Discussion

The prediction of the RNA secondary structure has gradually fallen into a bottleneck in traditional algorithm research over the past 40 years. In addition, few methods can be used to predict the RNA secondary structure with pseudoknots at present due to the complexity of traditional algorithms. With the rapid development of deep learning and machine learning, the bottleneck problems can be solved one after another based on the corresponding traditional optimization algorithms. The problem of the RNA secondary structure prediction with pseudoknots is no exception. In this article, a new type of deep learning prediction model named ATTfold was proposed based on the attention mechanism to predict the RNA secondary structure with pseudoknots. Our method was mainly divided into three parts, namely, Encoder-Decoder-Constrain. The Encoder part encoded the RNA sequence through a transformer model on the basis of the attention mechanism to obtain the hidden features. The Decoder part decoded the hidden features through a convolutional neural network to form an unconstrained base pairing score matrix. The Constrain part constrained the bases pairing scoring matrix, which was a condition with which the RNA sequence must comply with to form a secondary structure and was referred to as the hard constraint. In the end, the real and effective RNA secondary structure was predicted.

Comparatively speaking, the ATTfold method exhibited the following innovative points. First, it used the transformer model with a dominant position in the NLP direction to extract the hidden features of the RNA sequence. Furthermore, combined with the convolutional neural network, the two-dimensional matrix was decoded to form a pairing matrix. Second, the hard constraints were simplified, and the algorithm was optimized through the Lagrange Multiplier Method. Compared with the DP algorithm, the computational complexity of the algorithm was greatly reduced. Finally, the most important thing was how to predict RNA sequences with pseudoknots. Different from the traditional dot bracket representation method, whose one-dimensional structure only expresses the structure without pseudoknots, more different types of brackets should be added to express the structure with pseudoknots (Wang et al., 2019). ATTfold, on the other hand, used a two-dimensional symmetric matrix to show the true pairing of each base, thus displaying the real RNA secondary structure with pseudoknots.

According to the above results, under the training of tens of thousands of data, ATTfold was greatly improved compared with the traditional algorithms, but it also revealed some problems. When predicting short RNA sequences without pseudoknots, the average score was above 90%. For long RNA sequences with pseudoknots, the effect of our algorithm was inferior to short sequence prediction, even though it was superior to the traditional algorithms. Two causes may be responsible for this result. First, the most important issue for deep learning is the volume of data. Short RNA sequences account for over 80% of the total RNA data volume, and a long time is required to train against these short RNA sequences when training the network; by contrast, the proportion of long RNA sequences is very small. Second, the secondary structure of the long RNA sequences is more complicated, and it is more difficult for the models to extract hidden features. Consequently, more data are needed to fit the long RNA sequences well.

Collectively, findings in this study show that the new deep learning prediction model ATTfold, constructed based on the attention mechanism, has been qualitatively improved compared with the traditional algorithm, which can learn similar hidden features through RNA sequences of different families thereby predicting the secondary structure of different RNA families. Moreover, with the discovery of more long RNA sequences and their base pairing structures in the future, the overall performance of the ATTfold algorithm for multiple RNA families will be further improved. However, as far as the current data volume is concerned, although our algorithm has greatly improved the secondary structure prediction of long RNA sequences based on traditional algorithms, its accuracy is still relatively low. Therefore, when there are not enough long RNA sequences with known structures, more effective methods are warranted for prediction.

## Data Availability Statement

The original contributions presented in the study are included in the article/supplementary materials, further inquiries can be directed to the corresponding author/s.

## Author Contributions

YL and LD conceived and directed the project. YW designed the study and collected the raw data. YW and YG wrote the article and coded the procedure. HZ and SW revised the article critically for important intellectual content. HZ and ZL reviewed the data and the article. All the authors helped in drafting and reviewing the article before approving it for publication.

## Conflict of Interest

The authors declare that the research was conducted in the absence of any commercial or financial relationships that could be construed as a potential conflict of interest.
